# Gradient Boosting as a SNP Filter: an Evaluation Using Simulated and Hair Morphology Data

**DOI:** 10.4172/2153-0602.1000143

**Published:** 2013-10-20

**Authors:** GH Lubke, C Laurin, R Walters, N Eriksson, P Hysi, TD Spector, GW Montgomery, NG Martin, SE Medland, DI Boomsma

**Affiliations:** 1 Department of Psychology, University of Notre Dame, Notre Dame, IN, USA; 2 23 and Me, Inc., Mountain View, CA, USA; 3 Twin Research and Genetic Epidemiology, Genetic Epidemiologist, King's College London, London, England; 4 Genetic Epidemiology Laboratory, Queensland Institute of Medical Research, Brisbane, Australia; 5 Department of Biological Psychology, VU University Amsterdam, Amsterdam Netherlands

**Keywords:** GWAS, Boosting, GCTA

## Abstract

Typically, genome-wide association studies consist of regressing the phenotype on each SNP separately using an additive genetic model. Although statistical models for recessive, dominant, SNP-SNP, or SNP-environment interactions exist, the testing burden makes an evaluation of all possible effects impractical for genome-wide data.

We advocate a two-step approach where the first step consists of a filter that is sensitive to different types of SNP main and interactions effects. The aim is to substantially reduce the number of SNPs such that more specific modeling becomes feasible in a second step. We provide an evaluation of a statistical learning method called “gradient boosting machine” (GBM) that can be used as a filter. GBM does not require an a priori specification of a genetic model, and permits inclusion of large numbers of covariates. GBM can therefore be used to explore multiple GxE interactions, which would not be feasible within the parametric framework used in GWAS. We show in a simulation that GBM performs well even under conditions favorable to the standard additive regression model commonly used in GWAS, and is sensitive to the detection of interaction effects even if one of the interacting variables has a zero main effect. The latter would not be detected in GWAS. Our evaluation is accompanied by an analysis of empirical data concerning hair morphology. We estimate the phenotypic variance explained by increasing numbers of highest ranked SNPs, and show that it is sufficient to select 10K-20K SNPs in the first step of a two-step approach.

## Introduction

Genome-wide association (GWA) studies have a history of mixed successes. Although the investigation of numerous phenotypes has led to the detection of relevant single-nucleotide polymorphisms (SNPs), results of GWA studies fall short in explaining the genetic variance in phenotypes that is expected from twin and family studies [1-3]. Detection is challenging due to the fact that most effects are expected to be small. As Park et al. [4] have shown recently, the distribution of effect sizes might be best described by a mixture of two exponentials, which implies that the number of SNPs with small effect sizes is even higher than expected under a simple exponential relation between numbers of SNPs and effect size [4,5].

Furthermore, in a typical GWA study, a univariate phenotype is regressed on each SNP separately. Although simple statistical models are convenient in terms of computational ease and interpretability, these advantages are countered by potential loss of information. Most commonly only the additive genetic model is tested, and only few (if any) covariate main effects are included to maintain computational feasibility. This approach decreases the power to detect recessive or dominant effects, and excludes exploration of SNP-SNP and SNP-covariate interactions [6-10]. It is well-known that inclusion of relevant covariates in a regression analysis reduces error variance and thereby increases power. Furthermore, it might be of interest to include measures of comorbid disorders in GWAS of psychiatric disorders in order to assess genetic effects unique to the disorder, or to include proxies of environmental variables in order to assess conditional effects [11]. However, it is desirable to explore these effects without the need of formulating a specific model (e.g., additive and/or interaction effects).

Genome-wide detection studies might benefit from a two-step approach where the first step consists of substantially reducing the number of SNPs such that more complex models, or models permitting significance testing, can be applied in a second step. Desiderata for a first step filtering method include [1] methods have to be computationally attractive when applied to large, imputed data sets while providing sufficient sensitivity to detect small effects, [2] methods used in this step should be agnostic with respect to the genetic model relating a SNP to the phenotype, and [3] should permit the inclusion of multiple covariates, again preferably without the need to a priori specify the interrelations between covariates, or between covariates and SNPs. If the two steps, SNP selection and multivariate analysis with the selected SNPs, are carried out in separate samples, then the significance level in the second step depends on the number of selected SNPs corrected for the fact that tests are correlated [12,13]. A substantial reduction of the number of SNPs opens the door to fitting models in the second step that can be more complex both on the phenotype side (e.g., models that take into account interrelations between questionnaire items or symptom endorsements), and/or on the genotype side (e.g., interaction terms).

### Statistical learning methods

Statistical learning (SL) methods are designed to find important predictors in large data sets, and can be applied in cases where the number of predictors is much larger than the number of subjects. Different learning methods such as Random Forests and Bayesian Lasso have recently been applied to genome-wide data [14-19].

The three main differences between standard GWA studies and SL approaches are (1) that in the regression model commonly used in GWAS the association between a SNP and the phenotype is tested separately for each SNP whereas in SL all SNPs are analysed jointly,(2) in standard GWAS a specific genetic model is specified a priori that relates a SNP to the phenotype (e.g., additive, dominant, recessive) whereas SL models are built iteratively in a data driven fashion, and (3) the regression model used in standard GWAS is limited to SNP main effects whereas SL methods can capture SNP-SNP and SNP-covariate interactions.

**Figure 1:**
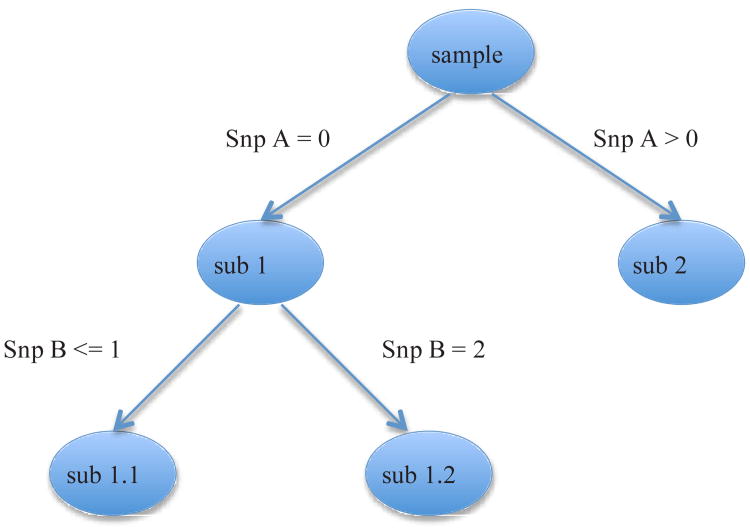
Results of GBM and additive GWA methods applied to hair morphology. At each split the sample is divided into subgroups based on an optimal cut point on the SNP with the best predictive performance.

We propose to use a statistical learning method called Gradient Boosting Machine (GBM) as the first step filter. The focus on the current paper is to provide an evaluation of GBM. GBM is a statistical learning method that fulfills the desiderata of a SNP selection method listed above. Previous research has shown that GBM performs as well or better than Random Forests (RF), a more well-known learning method [20]. We choose GBM as it has a much lower computational burden compared to RF, and is therefore more feasible in a genome-wide context. In pilot simulations RF took up to 10 times more time to complete without providing better detection rates in any of the settings. Different boosting algorithms have been proposed that utilize different optimization strategies [20-23]. In the current study we apply GBM which is implemented in the freely available R package gbm [24]. GBM builds a predictive model iteratively by adding “weak learners” [21-23]. The “weak learners” can be small regression or classification trees that are grown to a user- specified, usually small, number of splits. Figure 1 illustrates such a small regression tree. At each split a single SNP is selected to split the sample into two parts called “daughter nodes”. Given that SNPs can take values 0, 1, and 2, each SNP has two possible split points. The criterion to select a SNP and its split point is to achieve the best increase in homogeneity in the daughter nodes. At each iteration of the GBM algorithm, such a small tree is added to the model as a predictor, followed by searching for the next tree that optimally reduces misclassification (case/control phenotype) or residual (continuous phenotype). If trees consist of only a single split, then the resulting model is limited to main effects. Models where trees are grown to *k* splits can capture *k*-order interactions. All variables are considered at each step of the tree building algorithm in the search for the “best predictor”, that is, the variable that best increases homogeneity in the daughter nodes. If trees are grown to *k* splits, then the inclusion of covariates (e.g., environmental variables) results in an automatic search for conditional effects of SNPs and covariates.

GBM can be used to rank-order SNPs according to their cumulative predictive performance. The variable importance measure (VIM) used in GBM is similar to the Gini importance commonly used in Random Forests [25] VIMs for Random Forest have been reported to be biased for SNPs in LD [26-29]. Our own work showed a similar bias for the VIM used for GBM [30]. To correct for this bias, we have developed a sliding window algorithm that creates a large number of overlapping subsets of SNPs from a genome-wide data set [30]. For this study, the correlation between SNPs within subsets was set to not exceed 0.1, meaning that SNPs in higher LD were assigned to different subsets. The subsets were analyzed in parallel on a grid, followed by an aggregation of results over the subsets. The algorithm and its performance have been described in Walters et al. [30]. In addition to removing bias in importance measures due to LD, the algorithm makes statistical learning methods such as GBM computationally more feasible for genome-wide analyses. For instance, in the empirical analysis described below individual subsets comprise on average only 25K SNPs, which can be analyzed in approximately 3.5 hours. The computation time of the complete analysis depends on the number of available nodes in the grid.

### Evaluation of GBM

The main goal of the study is to evaluate the performance of GBM as a filter. We compare the sensitivity of ranking SNPs by p-value resulting from fitting the standard additive GWA model to Manolio et al. [1] ranking SNPs by p value resulting from a model that takes into account possible recessive and dominant effects [7], and Eichler et al. [2] to ranking SNPs using GBM. The comparison is carried out for simulated additive effects as well as interaction effects.

### Empirical study of hair morphology

Previous GWA studies of hair morphology have shown large as well as small and suggestive effects, making hair morphology a highly suitable phenotype for a comparison of GBM and standard GWA using empirical data. Hair curliness in Europeans varies widely, with 45% of northern populations having straight hair compared to 40% with wavy and 15% with curly hair [31]. A previous GWAS showed a robust effect of four single nucleotide polymorphisms (SNPs, rs17646946, rs11803731, rs4845418, rs12130862) in high LD (r^2^>.95) on chromosome 1 that explained approximately 6% of the variance of a normally distributed liability underlying the observed 3-category hair curliness (straight, wavy, curly) [32]. This large effect was replicated in a second adult and an adolescent family sample, and it was also found in an independent study examining a range of different phenotypes [33] Rs11803731 is located in the TCHH region (1q21). TCHH is expressed at high levels in the hair follicle, and mutations in rs11803731 might be related to structural variation of the trichohyalin protein [34-37]. In addition to the signal in the TCHH region, rs7349332 located in an intron of WNT10A on chromosome 2 (2q35) reached genome-wide significance in the study by Eriksson et al. [33] and was reported as a suggestive effect in Medland et al. [32] (p-value 1.36×10^−6^). Mutations in WNT10A are related to odonto-onycho-dermal dysplasia, characterized by symptoms including dry and misformed hair.

### Estimating a cutoff to select top ranked SNPs

We illustrate the SNP selection step using the empirical data of hair morphology by comparing GBM to GWA. To obtain an indication of how many SNPs to select in the first step, we use the program Genome-Wide Complex Analysis (GCTA) to estimate the phenotype variance that is explained by increasing percentiles of ranked SNPs compared to using all SNPs [38]. Again, we compare results based on rankings resulting from GBM and the standard GWA analysis.

## Materials and Methods

### Simulation study

Parametric models such as the regression model commonly used in GWA studies are unlikely to be outperformed by alternative methods if the parametric model is the correct model for the data. The weakness of fitting an a priori specified model lies in the potential for misspecifications and omissions of effects (e.g. recessive, dominant, SNP- SNP, and/or SNP-environment effects). However, since twin and family studies indicate that for many phenotypes a large part of the genetic effects are in fact additive (but see [10]), filtering methods aiming at reducing the number of SNPs should not underperform a selection based on ranking SNPs by p-value resulting from a standard GWA approach. Therefore, our simulations include data generated under the best-case scenario for standard GWA methods, as well as settings that we expect GBM to outperform other methods. The simulation consisted of two parts. For both parts we embedded simulated SNPs with specified minor allele frequency and effect size in a set of empirical genetic data consisting of 3000 SNPs observed in N=2000 subjects. The advantage of embedding simulated SNPs in empirical genetic noise is that the linkage disequilibrium (LD) structure is not simulated but observed in real data, while characteristics of the simulated SNPs are fully controlled. The first part of the simulation aimed at evaluating the performance of GBM to detect additive genetic effects. Here, data were generated under the additive genetic model that is commonly fitted in a GWAS. One hundred phenotype data sets were generated for all scenarios. For the first part of the simulation only main effects were evaluated. A simulated SNP explained on average either 0.15%, 0.2%, or 0.3% of the phenotypic variance. The SNP had either MAF=0.5, or MAF=0.1. For the second part of the simulation, the data contained interaction effects. We included 2 different scenarios: (1) two SNPs interact, have MAF=0.5, and both main effects and the interaction explain 0.3% of the variance, (2) same as (1), but one of the SNPs had a zero main effect. In both parts of the simulation, the data sets were analyzed with three methods, (1) GWA using the standard additive genetic model, which provides p-values for additive effects, (2) GWA using Robust SNP, which provides p-values that account for additive as well as potential recessive or dominant effects [7], and (3) GBM, which provides variable importance rankings that account for additive, recessive, dominant, and interaction effects. The performance of Robust SNP is described by the developers [7], and is included here as an intermediate option to illustrate the effect of permitting additive, recessive, and dominant effects on statistical power.

The main meta-parameters of GBM were chosen based on cross validation that was carried out in a small number of cells of the simulation design. The specific settings were (1) number of trees=3000, interaction depth=5, and shrinkage=0.0001. To evaluate simulation results, SNPs were ranked according to p-value (methods 1 and 2), or VIM (method 3). We then calculated the median rank of the simulated SNPs across data sets, and transform the median ranks into percentiles.

### Empirical example

The main sample in the empirical illustration consisted of unrelated individuals selected at random from two adult family samples that comprised a total of 3894 individuals from 2447 families. Genotype data were collected in several waves on different platforms, requiring imputation to combine samples. The data collection, genotyping, and imputation are described in Medland et al. [32]. Quality control (QC) was carried out using Plink closely following steps and criteria described in Anderson et al. [40] Subjects were removed if expected proportion of alleles shared IBS was >0.185, and if the proportion of missing alleles was >0.01. The criteria for excluding SNPs were MAF <0.01, SNP missingness proportion >0.01, and deviation from Hardy-Weinberg Equilibrium with p-value <10^−6^. In addition, following the example of the International Schizophrenia Consortium [41], we performed a haplotype-based test for missingness of SNPs. We excluded any SNP with missingness that was significantly predicted by the two neighboring SNPs on either side using p<10^−10^. QC resulted in selecting 2,359,291 SNPs and N=2235 subjects for our main analyses.

## Results

### Simulation results

In the first part of the simulation, we applied GBM, Robust SNP, and a standard additive GWA model to a simulated SNP embedded in 3000 empirical SNPs unassociated with the phenotype. The simulated SNPs explained 0.15, 0.20, or 0.30% of the phenotypic variance, respectively, and had either MAF=0.5 or MAF=0.1.

Table 1 shows the results of the first part of the simulation that evaluated detection of main effects. Results are presented as median rankings of the three simulated SNPs across simulation repetitions. Note that the phenotype data were generated under the additive GWAS model. As can be seen in Table 1, there are no differences between the three methods when MAF=0.5, showing that even under conditions that are optimal for additive GWAS, the GBM does not underperform. For MAF=0.1, there is trend for a decrease in performance of GBM as effect size decreases. Methods described in Walters et al. reduce the effect of MAF on GBM performance, and are currently investigated in a separate study.

The second part of the simulation demonstrates the advantage of GBM in the presence of SNP-SNP interaction effects (Table 2). The results demonstrate that GBM can detect SNPs with zero main effects in case the interacting variable has a small main effect. The zero effect SNP had a median rank within the 19th percentile when using GBM. As expected, fitting an additive GWA model resulted in chance detection for this SNP. Although in our simulation the interacting variable had the characteristics of a SNP (i.e., a random variable valued {0,1,2}, with MAF 0.5), these results can be generalized to interactions between SNPs with zero main effect and covariates such as for instance an items or scales measuring comorbid disorders, or proxies of environmental variables. Our simulation shows that GBM can detect interacting variables even if they have zero main effect as long as one of the interacting variables has a small main effect. Clearly, such effects can only be detected in a standard regression if they are explicitly included in the tested model, which is not feasible when exploring a larger number of covariates in a genome-wide analysis.

### Empirical illustration

In the empirical illustration using the hair morphology data, we compare top ranked SNPs resulting from additive GWAS and GBM. To address the question what proportion of SNPs should be selected in a first step, we estimated the variance in hair curliness that is explained by increasing proportions of SNPs ranked by VIM compared to SNPs ranked by p-value. Using an additive genetic model in a GWAS reproduced previous results concerning rs17646946, rs11803731, rs4845418, and rs12130862 on chromosome 1. The four SNPs reached genome-wide significance with p-values 3.47×10^−13^, 2.97×10^−13^, 1.63×10^−11^, and 1.24×10^−11^, respectively. No other SNP passed the threshold of 5×10^−8^.

We applied GBM to the hair curliness data using settings that had cross validated adequately in previous analyses of empirical and simulated data of comparable size. The settings are (1) number of trees in prediction model=3000, (2) shrinkage=0.001, and (3) bag fraction=.5, and (4) interaction depth=1. GBM successfully reproduced the GWAS results of the large effects of rs17646946, rs11803731, rs4845418, and rs12130862 in the 1q21 region on chromosome 1. These four SNPs had the highest VIMs. Rs7349332 located in WNT10A on chromosome 2, which reached genome-wide significance in the study by Eriksson et al. [33] and was reported as a suggestive effect in Medland et al. [32] was ranked in the upper 0.012 percentile in the GBM results. The ranking for this SNP was 284.

**Figure 2:**
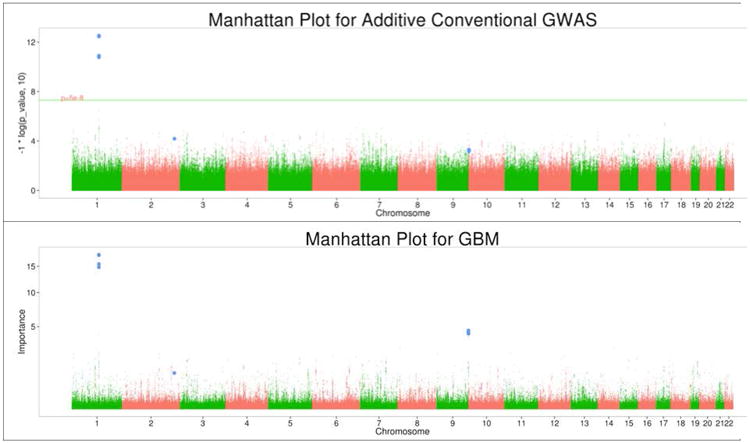
Manhattan plots for GBM, and GWAS. The green horizontal line marks the genome-wide significance level. Relevant SNPs on chromosomes 1, 2, and 8 are marked in blue.

The GBM results deviated from the additive genetic model results concerning the second largest signal detected with GBM (Figure 2). GBM implicated several SNPs in high LD on chromosome 9. These SNPs were ranked 5^th^-9^th^, that is, within the 0.000375^th^ percentile. To investigate whether the divergence between the additive genetic model and GBM regarding the signal on chromosome 9 was due to limiting the GWAS to an additive model, we tested rs2784081, which had the highest rank, in a dominant and a recessive genetic model. The recessive model resulted in β=0.89 (se=0.18), with associated p-value 5.89×10^−07^, whereas the dominant model was not significant (p-value 0.068). The additive model tested in the GWAS had a p-value of 5.08×10^−4^. Rs2784081 tags the tumor necrosis factor receptor-associated factor 2 gene, TRAF2, located at 9q34. TRAF2 is mainly known for its role in regulating programmed cell death (apoptosis).

Apoptosis in the hair follicle is described in detail in Botchkareva et al. [42]. The activity of the hair follicle is cyclic, and moves through phases of hair growth (anagen), apoptosis and hair loss (catagen), and a period of rest (telagen). Although TRAF2 plays a role in regulating the extrinsic apoptotic pathway in the hair follicle, no specific function of TRAF2 has been described that would more precisely help understand the association between TRAF2 and hair morphology.

We attempted to replicate the results concerning rs2784081 using two different independent data sets. The first data set was a sample of N=23,458 unrelated European individuals. The data stem from an ongoing web-based data collection, which is described in detail in Eriksson et al. [33] and which includes the data used in the Eriksson study as a subset. “European” in that sample is broadly defined, and includes individuals from multiple ethnic groups. Using the first 4 principal components to correct for stratification, rs2811761, which is in high LD (r^2^>.9) with rs2784081 was tested in an additive as well as a recessive model. Neither model resulted in statistical significance (Wald statistics<1.0, p values>.5). Narrowing the sample to Northern Europeans (N=13,605) in order to match population characteristics more closely to our original sample was equally unsuccessful.

The second data set used in our attempt to replicate the effect of rs2784081 consisted of participants of the Twins UK adult twin registry, which is a volunteer cohort of over 10,000 twins from the general population. The part of Twins UK with available genotype data is described in more detail in Hysi et al. [43]. We randomly selected one individual from each MZ pair (N=199), and included all non-MZ sibling pairs (N=192 pairs) and unrelated individuals (N=2049), resulting in a sample of N=2632 individuals. To account for clustering due to family structure caused by the non-MZ sib pairs we used Generalized Estimating Equations (GEE) to test for the association of rs2784081with binary hair curliness [44-46]. The recessive effect was estimated at β=0.417 with a standard error of se=0.210, resulting in a Wald statistic of 3.96 associated with p=0.047, thereby replicating the results of the main analysis. Additionally, we tested the additive genetic model. The allelic effect was smaller, β=0.143, but was associated with a smaller standard error, se=0.0696, thus producing a larger Wald statistic equaling 4.25, *p*=0.040.

### Estimating a cutoff for the selection of top SNPs

**Figure 3:**
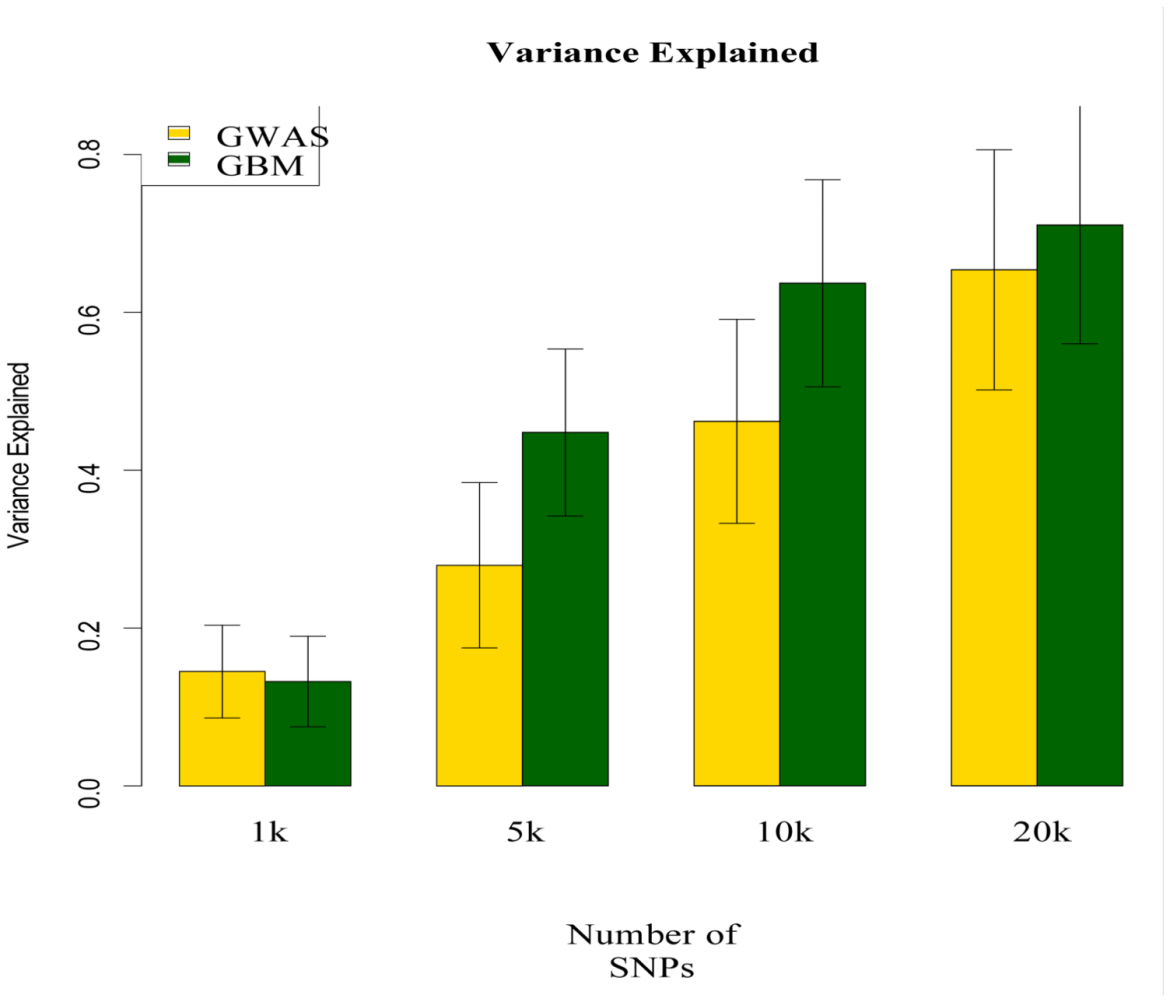
GCTA results Comparison of variance in hair curliness explained by top 1 k, 5 k, 10 k, and 20 k SNPs resulting from standard GWAS and GBM. Bars represent point estimates, whiskers 95% confidence intervals.

Selecting a proportion of top ranked SNPs in a first step filter provides the possibility of fitting more complex models in a second step. To obtain an indication which proportion of SNPs to select, and to further evaluate GBM as a first step filter we estimated how much variance in hair curliness was explained by the top 1k, 5k, 10k, and 20k of the ranked SNPs. The main objective of this part of our analysis was to obtain an indication of how many top ranked SNPs would be necessary to match the estimate of variance explained by all SNPs. This analysis was done using GCTA [38,47]. We compared the variance in hair curliness explained by the top ranked SNPs resulting from GBM to the same numbers of SNPs rank ordered by p-values resulting from the additive GWA model. The method implemented in GCTA consists of first computing the genetic similarity between all pairs of subjects using the observed SNP data. The second step of the method involves using the genetic similarity as a random effect to predict the phenotype in a linear mixed model. GCTA can be used for subsets of SNPs or all SNPs. Note that GCTA evaluates variance explained by additive effects. GCTA requires stringent QC especially when applied to case/control phenotypes [47]. In addition to an adaptation of the two-locus test to remove SNPs that controls for differential case/control calling errors, we included the first 4 principal component as covariates when estimating the phenotypic variance that is due to SNPs [48] Figure 3 shows point estimates and 95% confidence intervals corresponding to selecting increasing numbers of top ranked SNPs resulting from applying GBM and additive GWAS. In our analysis, the top 20K SNPs detected by GBM explain approximately the same variance as all SNPs in the data set. Furthermore, when comparing point estimates of SNPs ranked by GWAS and GBM, Figure 3 shows that these are very similar for 1000 SNPs, and higher for GBM than those of standard GWAS when considering the top 5K, 10K, and 20K. Confidence intervals overlap, however, and therefore results should be interpreted with caution. Obviously, both the ranking obtained through GBM, and the precision of the estimates of explained variance are, among others, dependent on sample size. The result that the top 20K of ∼2,4 million SNPs explain about the same variance in hair curliness highlights the potential of GBM as a first stage filter of genome-wide SNPs.

## Discussion

One of the advantages of GBM is that it is not necessary to specify a genetic model a priori. As noted by Lettre et al. [6], limiting a GWA study to the additive genetic model performs poorly in case the correct model is recessive. Furthermore, testing all possible genetic models requires appropriate adjustments for multiple testing [7]. A second advantage is the detection of interaction effects, not only between SNPs, but also between SNPs and included covariates, without the need to specify exactly which main or interactions effects to include in the model. The simulations showed the advantage of boosting over conventional methods is especially clear if one of the interacting variables has no main effect. Gradient boosting might therefore be especially interesting for the exploration of GxE interactions. As mentioned by Walters et al., adding several hundred variables in a GBM analysis of genome-wide SNPs does not noticeably increase the computation time [30].

Note that it is possible to ft GBM models that differ with respect to the order of interaction (e.g., up to *k*^th^ order interactions). In practice, complex phenotypes are often measured with multiple items. It is easy to show that using a sum score as a proxy of a multidimensional phenotype can entail a considerable loss of information, and therefore loss of statistical power to detect genetic effects [49-51]. Current methods that permit flexible modeling of multivariate phenotypes while accounting for potential interactions between SNPs or SNPs and covariates are limited by computational demands. GBM could be used to obtain scale specific SNP selections by carrying out analyses on multiple items or scales while including the other scales as predictors. The selected lists can then be combined in second step analyses that permit more complex modeling of multivariate phenotypes.

The empirical illustration showed that GBM is not limited to the detection of additive effects, however, the lack of a solid replication is a reminder that the selected top SNPs will include false positives. It is therefore important to carry out the second step analyses of a two-step genome-wide analysis in an independent sample. The advantages of a 2-step procedure are that more complex modeling is computationally feasible, and that the significance level of the second step depends on the much smaller number of SNPs included in the second step, corrected for LD between the selected SNPs.

In conclusion, our simulation showed that GBM performs well even under conditions that are optimally favorable for additive genetic GWA methods, and outperforms standard methods in case interactions are present. Furthermore, the illustration showed that GBM replicated previous GWAS results concerning large effects of four SNPs in Trichohyalin gene in the 1q21 region. Also, SNPs tagging *WNT10A* that had reached genome-wide significance or were reported as suggestive effects in previous studies ranked in the upper 0.01 percentile when ranking SNPs according to predictive importance. While an alternative first step filter could use test all three univariate genetic models (additive, dominant, recessive) and correct for Type I error as described in So and Sham, GBM has the additional advantage of providing SNP rankings that do not exclude epistatic effects, and that can include main and interaction effects involving large numbers of potentially interesting covariates.

## Figures and Tables

**Table 1:** Results of three simulated SNPs using GBM, Robust SNP, and a standard additive GWA model.

	MAF=0.5			MAF=0.1		
	0.15%	0.20%	0.30%	0.15%	0.20%	0.30%
GBM	5.6 (8.0)	3.6 (5.2)	1.6 (2.4)	19.6 (28.2)	9.8 (14.2)	2.0 (2.9)
RobustSNP	6.7 (9.4)	4.1 (6.1)	1.7 (2.4)	13.2 (18.9)	4.9 (7.2)	1.2 (1.8)
GWA	5.2 (7.6)	5.2 (7.7)	1.6 (2.3)	11.0 (14.3)	3.2 (4.8)	1.1 (1.5)

Note: Results are presented as percentile median ranks. For instance, averaged over Monte Carlo replications, 50% of the time a SNP explaining 0.15% of phenotypic variance is ranked within the 5.6 percentile. A robust measure of variability (Median Absolute Deviation, MAD) is given between brackets

**Table 2:** Results of two interacting simulated SNPs using GBM, Robust SNP, and a standard additive GWA model.

	*Scenario1 0.30/0.30*		*Scenario2 0.30/0*	
	SNP 1	SNP 2	SNP 1	SNP 2
GBM	1.3 (1.9)	0.8 (1.2)	1.1 (1.7)	19.0 (19.9)
RobustSNP	3.0 (4.2)	2.2 (3.3)	1.1 (1.6)	56.9 (33.7)
AdditiveGWA	1.7 (2.5)	1.8 (2.7)	0.8 (1.1)	52.1 (35.3)

Note: Results are presented as percentile median ranks of detecting SNP. A robust measure of variability (Median Absolute Deviation, MAD) is given between brackets. SNP1 always explains 0.3% of the variance, SNP2 either explains 0.3% (scenario1) or zero% (scenario2). Using GBM, a SNP with a zero main effect (SNP2, scenario2) is within the19th percentile in 50% of the Monte Carlo replications. Robust SNP and additive GWAS perform (as expected) only at chance level (i.e., median percentile rank around 50).
